# Hydrogen Sulfide Regulates the [Ca^2+^]_i_ Level in the Primary Medullary Neurons

**DOI:** 10.1155/2016/2735347

**Published:** 2016-10-20

**Authors:** Xiaoni Liu, Nana Zhang, Yingjiong Ding, Dongqing Cao, Ying Huang, Xiangjun Chen, Rui Wang, Ning Lu

**Affiliations:** ^1^Department of Physiology and Pathophysiology, Shanghai Medical College, Fudan University, Shanghai 200032, China; ^2^Department of Neurology, Huashan Hospital, Fudan University, Shanghai 200040, China; ^3^Department of Neurosurgery, Huashan Hospital, Fudan University, Shanghai 200040, China; ^4^The Cardiovascular and Metabolic Research Unit, Laurentian University, Sudbury, ON, Canada P3E 2C6

## Abstract

In the present study, we attempted to elucidate mechanisms for the regulation of intracellular calcium levels by H_2_S in primary rat medullary neurons. Our results showed that NaHS significantly increased the level of [Ca^2+^]_i_ in rat medullary neurons in a concentration-dependent manner. L-Cysteine and SAM significantly raised the level of [Ca^2+^]_i_ in the medullary neurons while HA and/or AOAA produced a reversal effect. In addition, L-cysteine and SAM significantly increased but HA and/or AOAA decreased the production of H_2_S in the cultured neurons. The [Ca^2+^]_i_ elevation induced by H_2_S was significantly diminished by EGTA-Ca^2+^-free solutions, and this elevation was also reduced by nifedipine or nimodipine and mibefradil, suggesting the role of L-type and/or T-type Ca^2+^ channels. Moreover, the effect of H_2_S on [Ca^2+^]_i_ level in neurons was significantly attenuated by BAPTA-AM and thapsigargin, suggesting the source of Ca^2+^. Therefore, we concluded that both exogenous and endogenous H_2_S elevates [Ca^2+^]_i_ level in primarily cultured rat medullary neurons via both increasing calcium influx and mobilizing intracellular Ca^2+^ stores from ER.

## 1. Introduction

Hydrogen sulfide (H_2_S) is an important gasotransmitter along with nitric oxide (NO), carbon monoxide (CO), and ammonium in addition to its conventional toxicological profile [1–3]. The endogenous production of H_2_S in the human body is catalyzed by several enzymes, including two pyridoxal-5′-phosphate- (PLP-) dependent enzymes, namely, cystathionine-*β*-synthase (CBS) and cystathionine-*γ*-lyase (CSE), and 3-mercaptopyruvate sulfurtransferase (MST) [4]. L-Cysteine and homocysteine or their derivatives are the substrates of these H_2_S-generating enzymes [4]. The expression of these H_2_S-generating enzymes is tissue specific. CBS is highly expressed in the central nervous system (CNS). CSE is mainly expressed in the cardiovascular system [2] and MST is considered as another source of H_2_S in the brain [5]. The physiological concentration of sulfide in brain tissue is detected to be 50~160 *μ*M [6], whereas the blood level of H_2_S is estimated at low micromolar to high nanomolar range [7].

H_2_S is a signaling molecule for neurotransmission and neuromodulation and is involved in learning, memory, and nociception [3]. H_2_S has been reported to enhance the induction of long-term potentiation (LTP), a synaptic model in learning and memory, and increase the sensitivity of NMDA receptor mediated response, indicating a neuroprotective effect of H_2_S on homocysteine-induced cerebrovascular pathology [8, 9]. NaHS attenuated the inflammation induced by LPS in microglia via inhibiting of p38-MAPK [10], suggesting the implication of H_2_S in the neuroprotection or treatment of cerebral ischemia and neuroinflammatory diseases. It was also found that H_2_S decreased blood pressure in various models of hypertension [11–14]. H_2_S treatment reduced blood pressure and oxidative stress in angiotensin II induced hypertensive mice [15] and in spontaneously hypertensive rats (SHRs) [14]. These findings indicate that H_2_S, as an important neuromodulator, produces antineuroinflammatory, antioxidant, and antiapoptotic effects in neurons and glial cells. However, the underlying mechanisms are still unsettled.

Calcium is second messenger for neuronal functions, such as release of neurotransmitters [16], synaptic plasticity [17, 18], neuronal excitation, and gene transcription [16]. Changes of intracellular free Ca^2+^ concentration ([Ca^2+^]_i_) may directly alter neuronal excitability [19]. It was reported that H_2_S increased [Ca^2+^]_i_ and induced Ca^2+^ waves in primary cultures of astrocytes [20] and regulated calcium homeostasis in microglial cells [21]. Another study showed that H_2_S modulated calcium homeostasis in cultured rat cerebellar granule neurons (CGN) as it induced activation of Ca^2+^ entry through L-type Ca^2+^ channels and thereby of neuronal activity [22]. It has also been reported that H_2_S increased [Ca^2+^]_i_ in SH-SY5Y neuronal cells by increasing Ca^2+^ influx via plasma membrane and the subsequent release of calcium from intracellular calcium store [19]. However, the regulation of H_2_S on [Ca^2+^]_i_ in the medullary neurons has not been demonstrated.

We recently reported that H_2_S exerts its cardiovascular effects by decreasing oxidative stress via inhibition of NADPH oxidase activity in the rostral ventrolateral medulla (RVLM) of SHRs [14]. RVLM, where sympathetic premotor neurons are located, is connected with other cardiovascular nuclei in the CNS, functioning to regulate the cardiovascular effects by regulating sympathetic nerve activity [23–25]. However, the molecular mechanisms for the neuromodulatory effect of H_2_S on RVLM are not clear. In the present study, we used the primarily cultured medullary neurons to investigate the effects of H_2_S on the level of [Ca^2+^]_i_. Our study provides the evidence that H_2_S increases [Ca^2+^]_i_ in neurons through several different mechanisms.

## 2. Materials and Methods

### 2.1. Chemicals

DMEM/F-12, neurobasal medium, and B27 supplement were obtained from Gibco Invitrogen Corporation (Carlsbad, CA, USA). Fura-2 AM and BAPTA-AM were obtained from Dojindo Molecular Technologies, Inc. Sodium hydrosulfide hydrate (NaHS), EGTA, thapsigargin (TG), nifedipine, nimodipine, and mibefradil were obtained from Sigma-Aldrich. Fura-2 AM and TG were dissolved in dimethyl sulfoxide (DMSO). The final concentration of DMSO did not exceed 0.1%. Anti-MAP-2 antibody produced in mouse, anti-MAP-2 antibody produced in rabbit, anti-CBS antibody produced in mouse, and anti-MST antibody produced in rabbit were obtained from Abcam. Anti-glutamate antibody produced from rabbit was obtained from Sigma-Aldrich. FITC goat anti-rabbit IgG (H+L) and Cy3 goat anti-mouse IgG (H+L) were obtained from Beyotime Biotechnology. Alexa Fluor® 488 goat anti-rabbit IgG (H+L) and Alexa Fluor 594 goat anti-mouse IgG (H+L) were obtained from Invitrogen.

### 2.2. Cell Cultures

Primary cultures of medullary neurons were prepared from 14-day-old embryos of Sprague Dawley rats. The fetal rats were humanely taken out and killed by decapitation, and then brain slices containing the entire medullary were prepared. The dissected tissues were removed and transferred to D-hanks' solution containing (in g/L) 8.0 NaCl, 0.4 KCl, 0.134 Na_2_HPO_4_·12H_2_O, 0.06 KH_2_PO_4_, 0.35 NaHCO_3_, and 1 glucose, pH 7.2~7.4, and finally chopped. The tissues were then treated with 0.125% trypsin in D-hanks' solution for 10~12 min at 37°C and gently triturated using flame-polished Pasteur pipettes. Cell suspension was centrifuged for 8 min at 1000 ×g. Then the cell pellets were resuspended in Dulbecco's modified Eagle's medium (DMEM) and F-12 supplement (1 : 1) with 10% fetal bovine serum (FBS) before plating onto glass-bottomed dishes coated with poly-L-lysine (20 *μ*g/mL for 12~24 h) and kept at 37°C in 5% CO_2_ incubator. After overnight incubation in DMEM, the medium was changed to neurobasal medium (Gibco) containing 15 mM glucose supplemented with 2% B27, 2 mM glutamine, 10 *μ*g/mL penicillin, and 10 *μ*g/mL streptomycin. The culture medium for medullary neurons was changed every 48 h. Microscopically, glial cells were not apparent in medullary neurons employing this protocol. The neurons were maintained for 7–10 days in primarily culture until used for calcium imaging.

### 2.3. Immunofluorescence Staining and Laser Confocal Microscopy

The primarily cultured medullary neurons were washed three times with D-hanks' solution and then cells were fixed with 1 mL 4% paraformaldehyde (PFA) in 0.1 M sodium phosphate buffer (0.1 M PB; pH 7.4) for 20 min. Afterward, cells were blocked with 1 mL 5% fetal bovine serum (FBS) for 30 min after being washed three times with D-hanks' solution. Then, cells were incubated with primary antibodies, namely, anti-MAP-2 (mouse, 1 : 200) and anti-glutamate (rabbit, 1 : 100), anti-MAP-2 antibody (rabbit, 1 : 200) and anti-CBS (mouse, 1 : 100), anti-MAP-2 antibody (mouse, 1 : 200) and anti-MST (rabbit, 1 : 50), and anti-MAP-2 antibody (rabbit, 1 : 200) and anti-CSE (mouse, 1 : 50), for 1 h at 37°C, plus an additional 12 h at 4°C. On the next day, the cell were washed three times with D-hanks' solution and incubated with fluorescent secondary antibodies, namely, FITC goat anti-rabbit IgG (H+L) (1 : 100) and Cy3 goat anti-mouse IgG (H+L) (1 : 100) or Alexa Fluor 488 goat anti-rabbit IgG (H+L) (1 : 500) and Alexa Fluor 594 goat anti-mouse IgG (H+L) (1 : 500), for 1 h at 37°C. Then cells were incubated with DAPI for 5 min at 37°C after being washed three times with D-hanks' solution. Finally, 500 *μ*L D-hanks' solution was added to the cell dishes for confocal comicroscopy (Zeiss LSM510, Jena, Germany).

### 2.4. [Ca^2+^]_i_ Measurements

To determine the level of [Ca^2+^]_i_, neurons were loaded with Ca^2+^-specific dye Fura-2 by incubating with 2.5 *μ*M Fura-2/AM (Molecular Probes) in HBSS (140 mM NaCl, 5 mM KCl, 1 mM MgCl_2_, 2 mM CaCl_2_, 10 mM glucose, and 10 mM HEPES-NaHS [pH 7.3]) at 37°C for 30 minutes and subsequently washed three times with HBSS to remove the excess extracellular Fura-2/AM. [Ca^2+^]_i_ was expressed as the ratio (*R*) of emitted fluorescence corresponding to excitation wavelengths of 340 nm and 380 nm. *R*/*R*_0_ was applied to assess the change of [Ca^2+^]_i_ level, in which *R*_0_ represents the fluorescent signal before drug treatment and *R* represents the signal after drug treatment.

To identify the source of Ca^2+^, the increase in [Ca^2+^]_i_ induced by NaHS was determined in Ca^2+^-free HBSS containing the extracellular Ca^2+^ chelator EGTA (2 mM). In addition, separate cultures were treated with two selective L-type Ca^2+^ channel blockers, nifedipine (10 *μ*M) and nimodipine (10 *μ*M), and with the selective T-type Ca^2+^ channel inhibitor mibefradil (2 *μ*M). To determine the involvement of intracellular Ca^2+^ stores in neurons, the cultures were treated with the intracellular Ca^2+^-chelating agent BAPTA-AM (50 *μ*M) and thapsigargin (1 *μ*M) with NaHS (200 *μ*M) stimulation. Thapsigargin is known to release Ca^2+^ from the endoplasmic reticulum by inhibiting Ca^2+^ ATPase. All experiments were carried out at 37°C and were repeated 4~6 times using different batches of cells.

### 2.5. Cell Viability Assay

Cell viability was analyzed by using Cell Counting Kit-8 (CCK-8, obtained from Dojindo). CCK-8 allows sensitive colorimetric assays for the determination of cell viability in cell proliferation and cytotoxicity assays. Dojindo's highly water-soluble tetrazolium salt, WST-8, is reduced by dehydrogenase activities in cells to give a yellow-color formazan dye, which is soluble in the tissue culture media. The amount of the formazan dye, generated by the activities of dehydrogenases in cells, is directly proportional to the number of living cells. The primary medullary neurons were cultured in 96-well plates at a cellular density of 0.5 × 10^4^ cells/well. Cells were cultured for 6~7 days at 37°C in 5% CO_2_ incubator. On the following day, the cultured neurons were treated for 30 min with different concentrations of NaHS. Afterward, each well of plates was added to 10 *μ*L CCK-8 solution and then incubated at 37°C, 5% CO_2_ for 2 h. Subsequently, the cell viability was assessed by measuring the absorbance at 450 nm.

### 2.6. Measurement of H_2_S Production

H_2_S levels in primarily cultured neurons were measured according to previously described methods with some modifications [26]. Briefly, primarily cultured medullary neurons were homogenized in ice-cold Tris-HCl (100 mM, pH 8.5) followed by centrifugation at 12,000 ×g for 20 min at 4°C. Thirty *μ*L supernatant was incubated with 80 *μ*L monobromobimane (MBB) for 40 min on a shaker at room temperature. Reaction was terminated by adding 20% formic acid and the level of H_2_S was tested by Gas Chromatograph-Mass Spectrometer (GC-MS). Proteins in the supernatant were quantified using BCA reagent (Shen Neng Bo Cai Corp.). H_2_S concentrations were determined using a curve generated with sodium sulfide (0–40 *μ*M) standards, and the H_2_S concentrations in cultured cells were expressed as *μ*M. H_2_S concentrations in primarily cultured medullary neurons were divided by the protein concentrations and were expressed as *μ*mol/g of protein.

### 2.7. Statistical Analysis

Statistical significance was determined using independent *t*-test or one-way ANOVA followed by SPSS 19.0. Data are presented as mean ± SEM. Difference at the *P* < 0.05 level was considered statistically significant.

## 3. Results

### 3.1. CBS and MST Were Expressed in the Medullary Neurons

**Figure 1:**
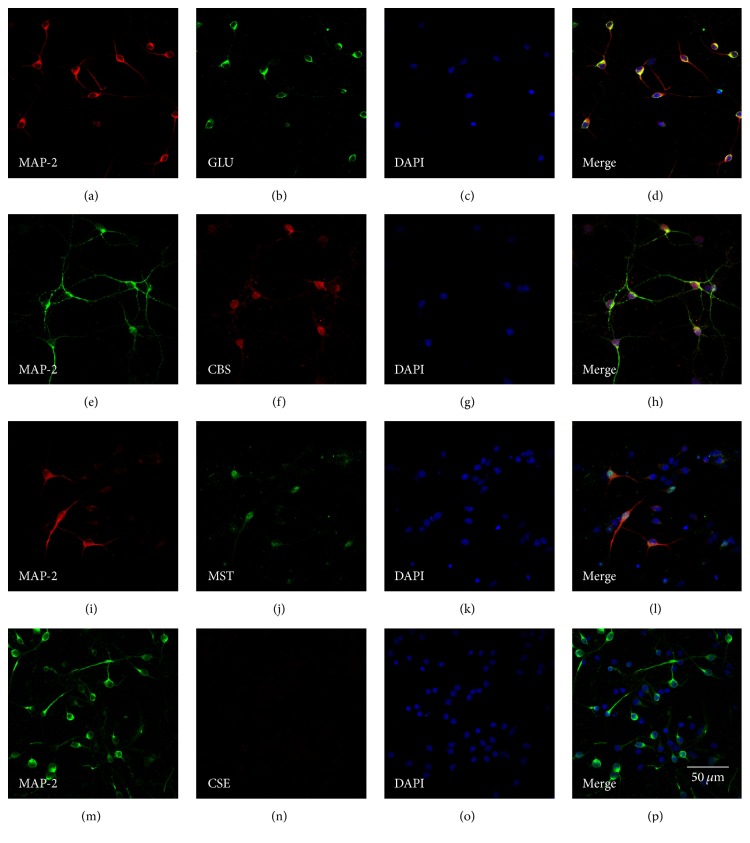
Identification of glutamate positive neurons ((a), (b), (c), and (d)) and the expression of CBS ((e), (f), (g), and (h)), MST ((i), (j), (k), and (l)), and CSE ((m), (n), (o), and (p)) in the rat primary medullary neurons. Confocal images showed that fluorescence micrograph of neurons stained with anti-MAP-2 antibody ((a), (e), (i), and (m), a neuronal maker), anti-glutamate antibody (b), anti-CBS antibody (f), anti-MST antibody (j), anti-CSE antibody (n), DAPI ((c), (g), (k), and (o)), or the merge of the other 3 photos ((d), (h), (l), and (p)). Scale bar = 50 *μ*m.

The primarily cultured medullary neurons were stained with anti-MAP-2 (red, Figure 1(a)) and anti-Glutamate (green, Figure 1(b)), suggesting that more than 90% of cells cultured were medullary neurons and most of them were glutamate positive neurons (Figure 1(d)).

Double-immunofluorescence labeling was used to study the specific localization of CBS in the primary medullary neurons. Fluorescence micrograph of neurons showed that neurons were stained with anti-MAP-2 antibody (green, Figure 1(e)). Anti-CBS was also stained with neurons (red, Figure 1(f)), and the merge of double-immunofluorescence labeling showed that more than 80% of neurons cultured were expressed with CBS (Figure 1(h)).

As MST is considered as another source of H_2_S in the brain [5], we also studied the expression of MST. Fluorescence micrograph of neurons showed that neurons were stained with anti-MAP-2 antibody (red, Figure 1(i)). Anti-MST was also stained with neurons (green, Figure 1(j)), and the merge of double-immunofluorescence labeling showed that MST was also expressed in the medullary neurons (Figure 1(l)).

As CSE is another mainly source of H_2_S, we also studied the expression of CSE. Fluorescence micrograph of neurons showed that neurons were stained with anti-MAP-2 antibody (green, Figure 1(m)), while anti-CSE was negatively stained with neurons (red, Figure 1(n)), and the merge of double-immunofluorescence labeling showed that CSE was negatively expressed in the primarily cultured medullary neurons (Figure 1(p)).

### 3.2. Effect of Exogenous H_2_S on [Ca^2+^]_i_ in the Primary Medullary Neurons

**Figure 2:**
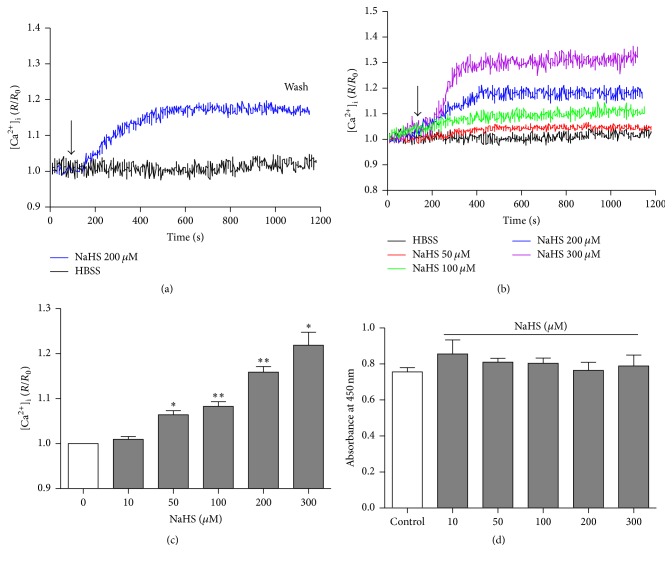
The effect of NaHS on [Ca^2+^]_i_ in the primarily cultured medullary neurons. (a) Typical elevation of [Ca^2+^]_i_ induced by NaHS at 200 *μ*M and washout of NaHS led [Ca^2+^]_i_ to decline. (b) Typical effects of different concentrations of NaHS (50, 100, 200, and 300 *μ*M) on [Ca^2+^]_i_. (c) Summary data of the peak increase in [Ca^2+^]_i_ in neurons stimulated with different concentrations of NaHS (50, 100, 200, and 300 *μ*M). ^*∗*^ *P* < 0.05; ^*∗∗*^ *P* < 0.01. (d) The cell viability analyzed by CCK-8 has no significance among different concentrations of NaHS. *n* = 6 in each group.

NaHS, a H_2_S donor, at 200 *μ*M induced a significant [Ca^2+^]_i_ increase in neurons and the effect of NaHS occurred rapidly and reached a peak within 10 min after administration. The level of [Ca^2+^]_i_ started to decline after washout (Figure 2(a)). NaHS (50–300 *μ*M) significantly increased the level of [Ca^2+^]_i_ in the medullary neurons in a concentration-dependent manner (Figures 2(b) and 2(c)). Moreover, the increase in [Ca^2+^]_i_ level induced by NaHS is persistent. We also examined cell viability after adding NaHS using CCK-8 assay. As shown in Figure 2(d), there was no significant difference in the cell viability between Control and the groups treated by NaHS at 50~300 *μ*M for 30 min. These data suggested that exogenous H_2_S could increase [Ca^2+^]_i_ level in medullary neurons, while the elevation of [Ca^2+^]_i_ induced by NaHS was not due to or caused by the cytotoxicity of NaHS.

### 3.3. Effect of Endogenous H_2_S on [Ca^2+^]_i_ in the Primary Medullary Neurons

**Figure 3:**
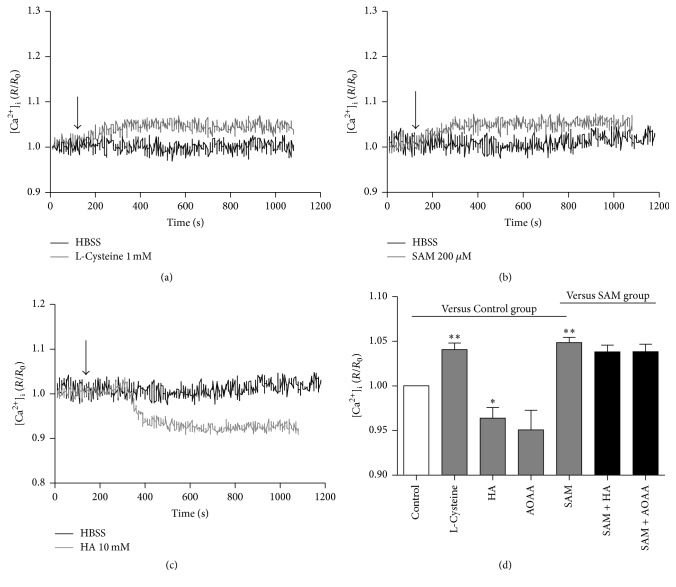
The effect of endogenous H_2_S on the [Ca^2+^]_i_ level in the primarily cultured medullary neurons. (a) Typical elevation of [Ca^2+^]_i_ induced by L-cysteine (the H_2_S precursor) at 1 mM. (b) Typical effects of SAM (an activator of CBS) at 200 *μ*M on [Ca^2+^]_i_ level. (c) Typical effects of HA (an inhibitor of CBS) at 10 mM on [Ca^2+^]_i_ level. (d) Group data showed the effects of endogenous H_2_S on the [Ca^2+^]_i_ level. AOAA (another inhibitor of CBS). ^*∗*^ *P* < 0.05, ^*∗∗*^ *P* < 0.01 versus Control group; *n* = 6 in each group.

**Figure 4:**
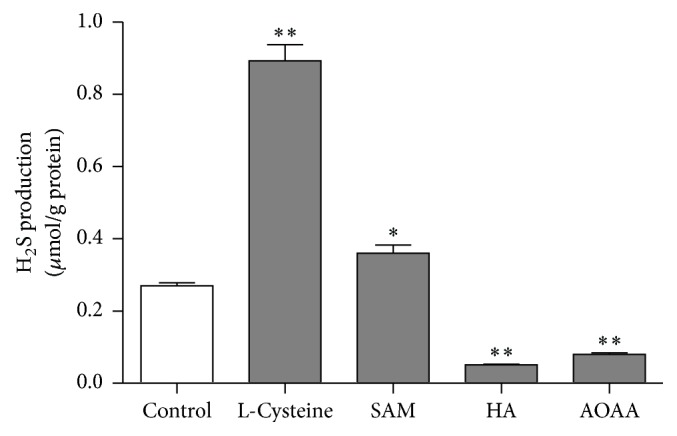
The production of H_2_S in the primarily cultured medullary neurons treated by L-cysteine (1 mM), SAM (200 *μ*M), HA (10 mM), and AOAA (10 mM), respectively. ^*∗*^ *P* < 0.05; ^*∗∗*^ *P* < 0.01. *n* ≥ 7 in each group.

To investigate the endogenous H_2_S  *n*  [Ca^2+^]_i_ in the primary medullary neurons, we applied L-cysteine (a substrate for H_2_S), SAM (a CBS activator), and HA and AOAA (two CBS inhibitors), respectively. The results showed that L-cysteine and SAM significantly raised the level of [Ca^2+^]_i_ in the medullary neurons (Figures 3(a) and 3(b)) while HA and/or AOAA produced a reversal effect (Figures 3(c) and 3(d)). And HA or AOAA can no longer inhibit [Ca^2+^]_i_ level in the presence of SAM. However, the stimulatory effect of SAM on [Ca^2+^]_i_ was not affected by HA or AOAA (Figure 3(d)). In addition, L-cysteine and SAM significantly increased but HA and/or AOAA (10 mM) decreased the production of H_2_S in the cultured neurons (Figure 4).

### 3.4. The Evolvement of L-Type and T-Type Ca^2+^ Channels in the Effect of H_2_S

**Figure 5:**
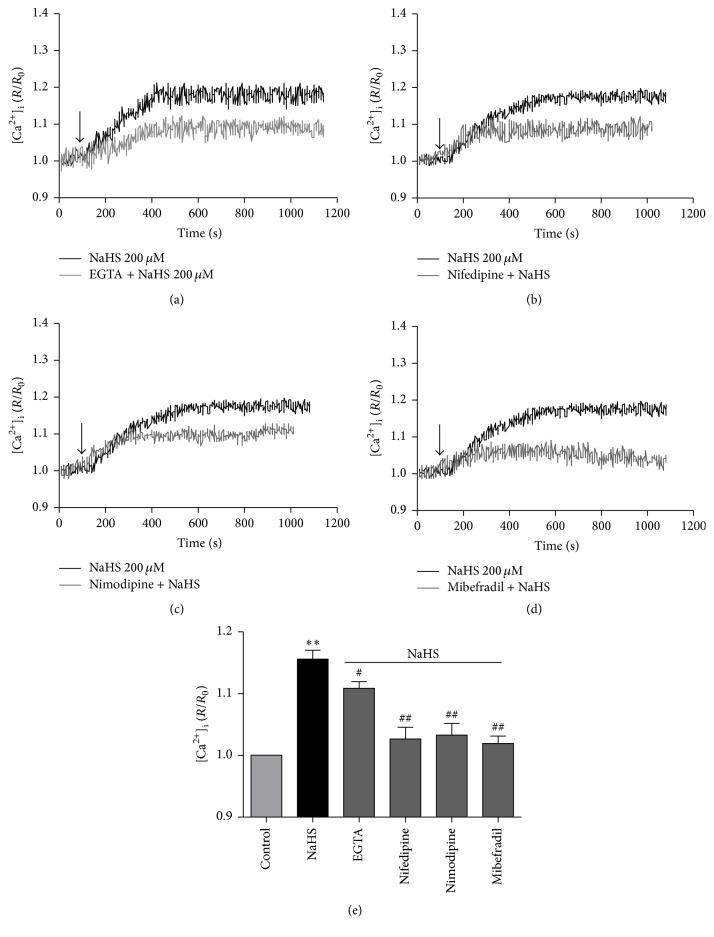
The effect of NaHS on [Ca^2+^]_i_ in the primarily cultured medullary neurons in Ca^2+^-free solution or in the presence of L-type and/or T-type Ca^2+^ channels inhibitors. ((a)–(c)) Typical suppression of [Ca^2+^]_i_ level induced by NaHS in EGTA-treated Ca^2+^-free solution (a) or in normal Ca^2+^ containing HBSS with or without nifedipine (10 *μ*M, (b)), nimodipine (10 *μ*M, (c)), and mibefradil (2 *μ*M, (d)). (e) Group data showed that the effects of NaHS were attenuated by EGTA-Ca^2+^-free solution and suppressed by nifedipine, nimodipine, or mibefradil. ^*∗∗*^ *P* < 0.01 versus Control; ^#^ *P* < 0.05 versus NaHS group; ^##^ *P* < 0.01 versus NaHS group. *n* = 5 in each group.

In order to determine the source of increased [Ca^2+^]_i_ in NaHS-stimulated medullary neurons, the cells were bathed either in Ca^2+^-free HBSS (containing 2 mM EGTA) or in normal HBSS. Similar to the data shown in Figures 2(a) and 2(b), NaHS at 200 *μ*M caused a robust increase in [Ca^2+^]_i_ within 10 min after incubation of normal Ca^2+^ containing HBSS (Figure 5(a)). This effect was partly abolished in cells bathed in Ca^2+^-free HBSS (Figures 5(a) and 5(e)).

As shown in Figures 5(b) and 5(c), both nifedipine (10 *μ*M) and nimodipine (10 *μ*M), two L-type Ca^2+^ channel blockers, significantly suppressed the effect of NaHS at 200 *μ*M (Figures 5(b), 5(c), and 5(e)). A selective T-type Ca^2+^ channel inhibitor, mibefradil (2 *μ*M), also inhibited the elevation of [Ca^2+^]_i_ level induced by NaHS (Figures 5(d) and 5(e))

### 3.5. The Involvement of Intracellular Ca^2+^ Stores in the Effect of H_2_S

**Figure 6:**
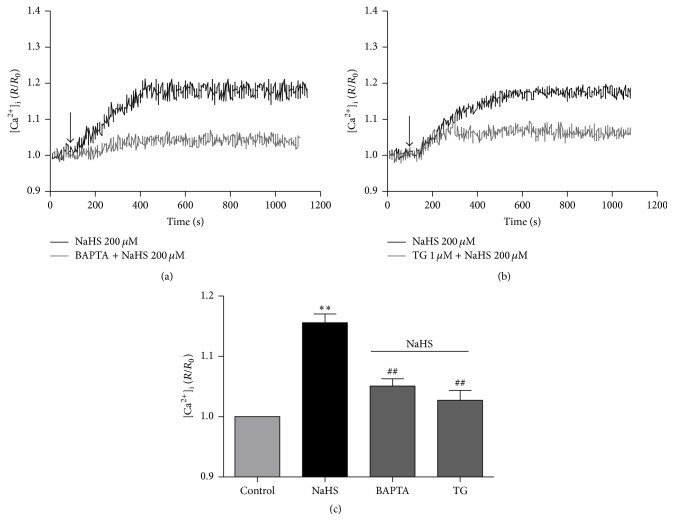
The effect of NaHS on [Ca^2+^]_i_ in the primarily cultured medullary neurons in the presence of intracellular Ca^2+^ chelator and sarco/endoplasmic reticulum Ca^2+^-ATPase blocker. ((a), (b)) Typical suppression of [Ca^2+^]_i_ level induced by NaHS with or without BAPTA-AM (50 *μ*M (a)) and thapsigargin (TG, 1 *μ*M (b)). (c) Group data showed that the effects of NaHS were suppressed by BAPTA-AM and thapsigargin. ^*∗∗*^ *P* < 0.01 versus Control; ^##^ *P* < 0.01 versus NaHS group. *n* = 5 in each group.

The neurons were treated with BAPTA-AM (an intracellular Ca^2+^ chelator) and thapsigargin (a sarco/endoplasmic reticulum Ca^2+^-ATPase blocker) with or without NaHS. The effect of NaHS on [Ca^2+^]_i_ level in neurons was significantly attenuated by depletion of BAPTA-AM (50 *μ*M, Figures 6(a) and 6(c)) and thapsigargin (TG, 1 *μ*M, Figures 6(b) and 6(c)).

## 4. Discussion

In the present study, we attempted to elucidate mechanisms for the regulation of intracellular calcium levels by H_2_S in primary rat medullary neurons. The results provide the evidence for the first time on the primarily cultured medullary neurons that H_2_S elevates [Ca^2+^]_i_ level via both increasing calcium influx and mobilizing intracellular Ca^2+^ stores from ER. Our conclusion is supported by the following findings: firstly, NaHS significantly increased the level of [Ca^2+^]_i_ in rat medullary neurons in a concentration-dependent manner. Secondly, L-cysteine and SAM significantly raised the level of [Ca^2+^]_i_ in the medullary neurons while HA and/or AOAA produced a reversal effect. In addition, L-cysteine and SAM significantly increased but HA and/or AOAA decreased the production of H_2_S in the cultured neurons. Thirdly, the Ca^2+^ elevation induced by H_2_S was significantly diminished by EGTA-Ca^2+^-free solutions, and this elevation was also reduced by nifedipine or nimodipine (an antagonist of L-type Ca^2+^ channel) and mibefradil (an antagonist of T-type Ca^2+^ channel), suggesting the role of L-type and/or T-type Ca^2+^ channels. Lastly, the effect of H_2_S on [Ca^2+^]_i_ level in neurons was significantly attenuated by BAPTA-AM (an intracellular Ca^2+^ chelator, 50 *μ*M) and thapsigargin (a sarco/endoplasmic reticulum Ca^2+^-ATPase blocker, 1 *μ*M), suggesting the source of Ca^2+^.

Previous studies have demonstrated the crucial role of H_2_S homeostasis in hypertension. Administration of H_2_S donors and precursors decreases mean blood pressure in various hypertensive models (chronic inhibition of nitric oxide synthase, two-kidney-one-clip, and SHRs) [11–13, 27]. One of the molecular targets for the cellular effect of H_2_S is K_ATP_ channels [28]. It has been reported that H_2_S in the RVLM inhibits sympathetic vasomotor tone through opening K_ATP_ channels [29, 30]. We have shown that H_2_S in RVLM suppressed the blood pressure in SHRs [14]. The signaling mechanisms in the CNS of the antihypertensive effect of H_2_S have not been elucidated. On the other hand, the antihypertensive effects of H_2_S in the caudal ventrolateral medulla (CVLM) involved K_ATP_ channels and glutamic acid receptor [31, 32]. Glutamate acid has been suggested to be an important neurotransmitter in antihypertensive effects of H_2_S. In order to provide more evidence for the regulation of H_2_S on the cardiovascular effects, we mainly focused on the medullary neurons in vitro. To ensure the cultured medullary cells were the target neurons which were glutamate positive neurons, we demonstrated that glutamate was coexpressed in more than 90% of primarily cultured medullary neurons. H_2_S is mainly produced endogenously by CBS and MST in the CNS [4, 5] and CBS is mainly expressed in the hippocampus and cerebellum, as well as the cerebral cortex and brain stem [8]. Our previous study has shown that CBS immunoreactivity was found in the rostral ventrolateral medulla (RVLM) neurons in vivo and the level of CBS proteins in the RVLM was lower in SHRs than in WKY rats [14]. In this study, our results showed that more than 80% of cultured medullary neurons were glutamate positive neurons, and CBS and MST were, respectively, expressed in these neurons in vitro, while the expression of CSE was negative in these primarily cultured medullary neurons. These results provided the basis of study of H_2_S.

Calcium plays an important role in regulating a great variety of neuronal processes such as release of neurotransmitters, synaptic plasticity, neuronal excitation, and gene transcription. It was reported that H_2_S increased [Ca^2+^]_i_ in SH-SY5Y neuronal cells by increasing Ca^2+^ influx via plasma membrane and in turn releasing calcium from intracellular calcium store [19]. Exerting its function as a gasotransmitter, H_2_S regulates calcium homeostasis in neurons via both increasing calcium influx and mobilizing calcium from ER [19]. In CNS, H_2_S activates L-type Ca^2+^ channels in rat cerebellar granule neurons to increase calcium signals which were inhibited by nifedipine and nimodipine [33] and in hippocampal slices and microglia to induce hippocampal LTP and Ca^2+^ waves in astrocytes [20]. H_2_S also activates T-type Ca^2+^ channels in NG108-15 (neuroblastoma cell line) involved in neuronal differentiation [34] and activates TRPA1 channels in rat sensory neurons from dorsal root ganglion [35]. The regulation of [Ca^2+^]_i_ in the medullary neurons by H_2_S has not been reported. Our results showed that NaHS (50–300 *μ*M) significantly increased the level of [Ca^2+^]_i_ in rat medullary neurons in a concentration-dependent manner. On the other hand, L-cysteine and SAM significantly raised the level of [Ca^2+^]_i_ in the medullary neurons while HA and/or AOAA produced a reversal effect. Meanwhile, HA or AOAA can no longer inhibit [Ca^2+^]_i_ level in the presence of SAM. However, the stimulatory effect of SAM on [Ca^2+^]_i_ was not affected by HA or AOAA. The possible reasons may be considered as follows: on the one hand, the affinity of SAM to CBS is much greater than that of HA or AOAA so that the stimulatory effect of SAM is dominating; on the other hand, the possibility that the increasing effects of L-cysteine and SAM on Ca^2+^ may be a H_2_S-independent manner is not ruled out and is needed to be addressed in the future. In addition, L-cysteine and SAM significantly increased the production of H_2_S in the cultured neurons and HA and/or AOAA decreased the production of H_2_S. These results suggested that both exogenous H_2_S and endogenous H_2_S increase the level of [Ca^2+^]_i_ in the medullary neurons.

[Ca^2+^]_i_ is controlled by Ca^2+^ channels in the membrane and intracellular Ca^2+^ stores [36]. The changes of [Ca^2+^]_i_ due to extracellular Ca^2+^ influx may be facilitated by voltage-gated channels, transmitter-gated Ca^2+^ permeant ion channels, transient receptor potential (TRP) ion channels, and Ca^2+^ pumps located in the plasma membrane [1, 36]. The function of status of intracellular Ca^2+^ stores is controlled by ryanodine receptor (RyR) channels, inositol triphosphate receptor (IP3R) channels, and sarcoendoplasmic reticular Ca^2+^ ATPases (SERCA) [1, 36]. In order to determine the source of [Ca^2+^]_i_ in NaHS-stimulated medullary neurons, we applied to the EGTA-Ca^2+^-free solution and BAPTA-AM to chelate the extracellular Ca^2+^ and intracellular Ca^2+^, respectively. The results showed that the Ca^2+^ elevation induced by H_2_S was significantly diminished by EGTA-Ca^2+^-free solutions and/or BAPTA-AM, suggesting that the elevation of [Ca^2+^]_i_ level in medullary neurons partly involved both an influx of extracellular Ca^2+^ and the intracellular Ca^2+^ stores. That thapsigargin (a sarco/endoplasmic reticulum Ca^2+^-ATPase blocker) significantly attenuates the effect of H_2_S on [Ca^2+^]_i_ level in neurons further suggests that NaHS releases calcium from intracellular Ca^2+^ stores.

In summary, the present study demonstrates that both exogenous H_2_S and endogenous H_2_S elevate [Ca^2+^]_i_ level in primarily cultured rat medullary neurons via both increasing calcium influx and mobilizing intracellular Ca^2+^ stores from ER.
